# Head-to-head comparison of MRI-based PI-RR scoring system and 68 Ga-PSMA-PET/CT: enhancing prostate cancer recurrence detection after radical prostatectomy

**DOI:** 10.1007/s11547-026-02265-2

**Published:** 2026-09-25

**Authors:** Ludovica Laschena, Martina Pecoraro, Emanuele Messina, Marco Bicchetti, Antonella Borrelli, Ailin Dehghanpour, Francesca Mezzapesa, Nicholas Landini, Giuseppe De Vincentis, Valeria Panebianco

**Affiliations:** Department of Radiological Sciences, Oncology and Pathology, Sapienza University of Rome, Rome, Italy

**Keywords:** Prostate cancer recurrence, MRI, PSMA-PET/CT, Radical prostatectomy, Prostate imaging for recurrence reporting (PI-RR) score

## Abstract

**Purpose:**

To compare the diagnostic performance of MRI using the PI-RR scoring system and PSMA-PET/CT in detecting prostate cancer (PCa) local recurrence after radical prostatectomy (RP), and to identify clinical and imaging variables independently associated with recurrence.

**Materials and Methods:**

This retrospective, multicenter study evaluated 119 patients with suspected PCa recurrence who underwent both MRI and PSMA-PET/CT; MRI was performed within a time window ranging from up to 3 months before to a maximum of 1 month after the PET/CT examination; the time range was defined within MDTs. Both radiologists and nuclear medicine physicians were blinded to clinical and opposite imaging data during image interpretation. Exclusion criteria included insufficient follow-up, recent pelvic radiation (within 6 months), and history of hormone therapy. Reference standard was defined using histopathologic findings, follow-up imaging, or clinical response to treatment. Sensitivity, specificity, PPV, NPV, and accuracy were calculated. The Cohen’s kappa coefficient was used to determine inter-reader agreement. Univariable and multivariable logistic regression analyses were performed.

**Results:**

MRI, considering a PI-RR ≥ 3, detected recurrence in 69 patients versus 48 with PSMA-PET/CT (*p* = 0.006). MRI showed significantly higher sensitivity (86%, 95% CI 75–92% vs. 71%, 95% CI 59–80%; *p* = 0.03) and a trend toward higher specificity (78%, 95% CI 65–87% vs. 52%, 95% CI 39–65%; *p* = 0.06), PPV (74% vs. 51%), NPV (89% vs. 73%). Overall accuracy was also superior for MRI (81%, 95% CI 74–87% vs. 63%, 95% CI 54–71%). ROC analysis confirmed a higher AUC for MRI (0.87, 95% CI 0.80–0.93) compared with PSMA-PET/CT (0.62, 95% CI 0.52–0.72; ΔAUC = 0.25, *p* < 0.001). Multivariable analysis showed that only age (*p* = 0.002) and PI-RR score ≥ 3 (*p* = 0.001) were independently correlated with recurrence. Postoperative risk grouping was correlated only at univariable analysis. Among the 119 included patients, histopathologic confirmation was available in 3% (*n* = 4), treatment response (either biochemical or imaging based) in 31% (n = 37), follow-up with imaging in 33% (*n* = 39), and clinical-laboratory follow-up in 33% (*n* = 39).The median follow-up was 12 months (IQR, 9–18). The effective recurrence rate was 58%.

**Conclusions:**

MRI demonstrated strong diagnostic performance in detecting prostate cancer local recurrence. These findings suggest that MRI may be particularly useful for rule out local recurrence in low-risk patients, while PET/CT could be more appropriate for rule in, both local and distant in higher-risk patients. However, this proposed risk-adapted imaging strategy should be considered hypothesis-generating and warrants further validation through prespecified risk-stratified analyses. Limitations include the retrospective design, the composite reference standard, and the relatively high recurrence prevalence, which may have influenced predictive values.

## Introduction

Prostate cancer (PCa) represents the most common cancer in men and the fifth leading cause of cancer-related mortality, with rising prevalence and mortality [1]. Possible PCa active treatments include radical prostatectomy (RP) or radiotherapy (RT), after which over half of patients experience biochemical recurrence (BCR) or PSA persistence [2, 3].

The definition of BCR is not standardized; however, it is commonly referred to as a prostate-specific antigen (PSA) of ≥ 0.2 in 2 consecutive measurements after RP, and PSA higher than 2 ng/mL compared to the nadir PSA after RT, and occurs in about 25% of patients treated with RP [4, 5]. PSA nadir is defined as the lowest PSA level reached after radiotherapy (RT) [6].

Moreover, Tilki et al. proposed a BCR risk classification, highlighting how patients with a Gleason score < 8 and a PSA doubling time > 1 year (after RP) and an interval to biochemical failure (IBF) > 18 months (after RT) should be considered at low risk; patients with a Gleason score 8–10 or a PSA doubling time ≤ 1 year (after RP) and an interval to biochemical failure (IBF) ≤ 18 months (after RT) should be considered at high risk [7].

On the other hand, PSA persistence is referred to as a detectable PSA of > 0.1 ng/mL within 4–8 weeks after surgery [2] (Suppl. Table 1). Depending on clinical needs and the type of primary therapy administered, the imaging strategy should be thorough, using several modalities to aid in both local and systemic staging.

Traditional imaging approaches, such as computed tomography (CT) and bone scintigraphy (BS) have demonstrated low accuracy for restaging patients with BCR [8, 9]. Conversely, magnetic resonance imaging (MRI) has shown high accuracy in the identification of prostate cancer recurrence in patients treated with whole-gland therapies, therefore after both RP and RT [10–13]. During the last decade, nuclear medicine techniques such as positron emission tomography (PET) with carbon 11(11C)-choline and fluorine 18(18F)-choline have proven to be more accurate than conventional diagnostic modalities for restaging patients experiencing PSA increase, allowing the differentiation of local and systemic recurrence [14, 15]. However, use of Gallium 68(68 Ga)-prostate-specific membrane antigen (PSMA) as a tracer for PET/CT scans has shown even greater accuracy in the early diagnosis of recurrence when compared to 18F-choline PET/CT[11]. In recent years, PSMA-PET/CT has become essential for restaging patients with PSA-only recurrence, demonstrating remarkable sensitivity for detecting local recurrence after RP and for assessing systemic disease [13].

According to international guidelines, the optimal imaging strategy to evaluate BCR remains debated. The NCCN guidelines recommend PSMA-PET/CT or PET/MRI as the primary imaging tools for initial staging and biochemical recurrence, given their superior sensitivity and specificity over conventional imaging [16]. Conversely, the EAU guidelines emphasize the role of MRI in detecting local recurrence after RT [2]. This lack of consensus underscores the need to clarify the optimal imaging approach and to assess the complementary role of MRI and PSMA-PET/CT in patients with BCR.

Moreover, since the development of the Prostate Imaging for Recurrence Reporting (PI-RR) score, MRI has been shown to be accurate in the early detection of PCa local recurrence, after both RT and RP [17–19]. The PI-RR score aims to standardize image acquisition, interpretation and reporting after RT and RP, providing a 5 point assessment scale for predicting the likelihood of PCa recurrence, combining criteria from multiple MRI techniques [17, 20]. The PI-RR scoring system is grounded in both anatomical and functional imaging parameters, analogous to the PI-RADS framework [21]. Anatomical assessment is based on T2-weighted imaging (T2WI), whereas functional evaluation reflects tissue cellularity and vascularity, assessed through diffusion-weighted imaging (DWI) and dynamic contrast-enhanced (DCE) sequences, respectively.

Additionally, the role of PI-RR scoring system has been currently validated in the literature, proving its reliable and robust role for the detection of local PCa recurrence [22–27].

While both MRI using the PI-RR scoring system and PSMA-PET/CT are established tools for detecting local prostate cancer recurrence, direct comparative evidence within the same patient cohort remains limited. Systematic head-to-head evaluation using standardized criteria is, therefore, warranted to better define their relative and complementary diagnostic value.

Based on this background, the primary aim of this study was to compare the performance of 68 Ga-PSMA-PET/CT and MRI using PI-RR score for the identification of local recurrence. Additionally, we investigated clinical and imaging variables that independently correlated with PCa recurrence as a secondary aim.

## Material and methods

### Patient population and study design

This observational study involved a retrospective analysis of a prospectively maintained cohort. The study met the ethical standards set by the Declaration of Helsinki, and all patients were fully informed about the study, providing written consent before participating. The Institutional Review Board and Local Ethical Committee (Territoriale Lazio Area 1) formally approved the study (Rif. CE n.7087 prot. n.146/2023), ensuring all ethical recommendations were followed.

Patients undergoing RP were enrolled from June 2021 to March 2023 and were offered MRI examination because of prostate cancer recurrence suspicion based on postoperative PSA value.

All the MRI examinations were performed within a time window ranging from up to 3 months before to a maximum of 1 month after the PET/CT examination, as defined in multidisciplinary teams (MDTs), in agreement with clinicians and radiologists.

All MRI examinations were performed at a single radiology center, while all PSMA-PET/CT studies were conducted at the same external nuclear medicine facility. Both imaging modalities followed standardized acquisition and reconstruction protocols using consistent scanner settings.

Exclusion criteria included insufficient follow-up data, pelvic radiotherapy performed within six months before radical prostatectomy, and a history of hormone therapy. Patients were excluded if prior treatments or incomplete clinical data could interfere with the assessment of true local recurrence or with the standardization of imaging interpretation. These criteria were applied to ensure a homogeneous cohort, as the PI-RR score is specifically designed for evaluating recurrence in patients treated exclusively with either RP or RT.

### MRI acquisition and reporting

Pelvic MRI was performed at 3.0 T (MAGNETOM Vida, Siemens Healthineers) with a 32-channel phased-array body coil. The MRI protocol was in accordance with the original PI-RR document’s recommendations [17] (Supplementary Table 2). Image analysis was performed by two experienced urogenital radiologists with more than 15 years and 4 years of experience in prostate imaging, respectively. Readers were blinded to clinic-pathological information, including the PSA trend and PET/CT results; they were only aware of the primary tumor location, which is necessary to assign PI-RR scoring. They evaluated the MRI scans independently, following PI-RR evaluation algorithm [17–19] and assigned scores to each MRI sequence based on specific PI-RR assessment categories in the post-RP setting; any discrepancies between the readers were resolved by consensus.

A PI-RR cutoff score = 3 (recurrence uncertain) or ≥ 4 (recurrence highly likely) was used to define a scan positive for local recurrence. For patients with more than one lesion, only the lesion with the highest PI-RR score was used for the analysis.

### PSMA-PET/CT acquisition and reporting

PSMA-PET/CT scans were acquired with a standard technique, in accordance with international procedural guidelines, using dedicated state-of-the-art scanner. All the patients underwent a 68 Ga-PSMA-PET/CT on different scanners, after a single injection of 68 Ga-PSMA-11 (mean dose 2 MBq/kg). A low-dose CT was acquired (with parameters of 120 keV and 80 mA) according to the European Association of Nuclear Medicine (EANM) procedure guidelines [28]. 68 Ga-PSMA-11 was synthesized in the involved center according to good manufacturing practices and in accordance with international guidelines.

All the scans were reported by two expert nuclear medicine physicians, experienced in PSMA-PET/CT oncologic imaging, blinded to MRI results. Based on procedure recommendations for PCa imaging, the investigations were classified as positive or negative in terms of identifying findings suggestive of PCa recurrence, and the anatomic site of recurrence was reported. The parameters used to classify PSMA-positive lesions as suspicious for PCa recurrence were in accordance with the existing literature [28, 29]. The main factor for the final diagnosis was visual interpretation. The maximum standardized uptake value (SUVmax) was calculated for all suspicious lesions, and the low-dose CT scan was used to examine the dimensions of abnormal findings.

### Definition of the reference standard

The criteria for establishing a true-positive PI-RR assessment were:

- Confirmation through histopathologic findings from the prostatectomy bed.
- Observation of a reduction in the size of a previously identified recurrence on images or a decrease in PSA levels following therapy (including both androgen deprivation therapy and salvage therapy) over a follow-up period of more than 24 months. This applied exclusively to patients with no signs of regional or distant metastasis as shown by nuclear medicine imaging (such as gallium PSMA or choline PET/CT scans and/or bone scans) at the time of the MRI.
- An increase in size observed during follow-up imaging (including pelvic MRI, gallium PSMA, or choline PET/CT) after more than 12 months.

The criteria for defining a true-negative finding included a negative result during follow-up imaging (including pelvic MRI, gallium PSMA, or choline PET/CT) conducted after at least 12 months, along with no increase in serum PSA levels for at least 24 months [25].

### Statistical analysis

Considering the study cohort (*n* = 119; 70 recurrence-positive and 49 recurrence-negative cases), with α = 0.05 and statistical power (1–β) = 0.80, the minimal detectable difference in AUC between MRI and PSMA-PET/CT was approximately 0.09. Therefore, the study was adequately powered to detect moderate-to-large differences in diagnostic performance between the two imaging modalities.

The performance of MRI and PSMA-PET/CT was assessed by means of receiver operating characteristic (ROC) curve analysis for the most experienced reader. For MRI, the area under the ROC curve (AUC) was calculated using a PI-RR cutoff ≥ 3. Sensitivity, specificity, positive predictive value (PPV), negative predictive value (NPV), and accuracy were computed for both MRI using the PI-RR score and PSMA-PET/CT.

Diagnostic accuracy comparisons between MRI and PSMA-PET/CT were performed using paired statistical analysis (McNemar test for correlated proportions). Inter-reader agreement was evaluated with Cohen’s κ statistics, including 95% confidence intervals (95% CI), for both PI-RR score assignment and PSMA-PET/CT assessment. The strength of agreement was interpreted according to the Landis and Koch benchmarks: κ < 0.00 = poor; 0.00–0.20 = slight; 0.21–0.40 = fair; 0.41–0.60 = moderate; 0.61–0.80 = substantial; and 0.81–1.00 = almost perfect agreement.

The correlation between patient demographics, clinical characteristics, and the presence of local recurrence was analyzed using the Pearson’s χ^2^ test. Univariable analysis included variables such as age, PSA values at diagnosis and at suspected relapse, Gleason score, International Society of Urological Pathology (ISUP) grade group, surgical margin status, biochemical recurrence (BCR) risk group according to EAU criteria [5], PI-RR score (as assigned by the most experienced reader), and PSMA-PET/CT results. Variables showing statistical significance in univariable analysis were entered into a multivariable logistic regression model to identify independent predictors of local recurrence. Age was treated as a categorical variable (< 70 vs ≥ 70 years).

All analyses were performed using SPSS software (version 25). A two-tailed significance level of *p* < 0.05 was used to determine statistical significance.

## Results

### Patient demographics

**Fig. 1:**
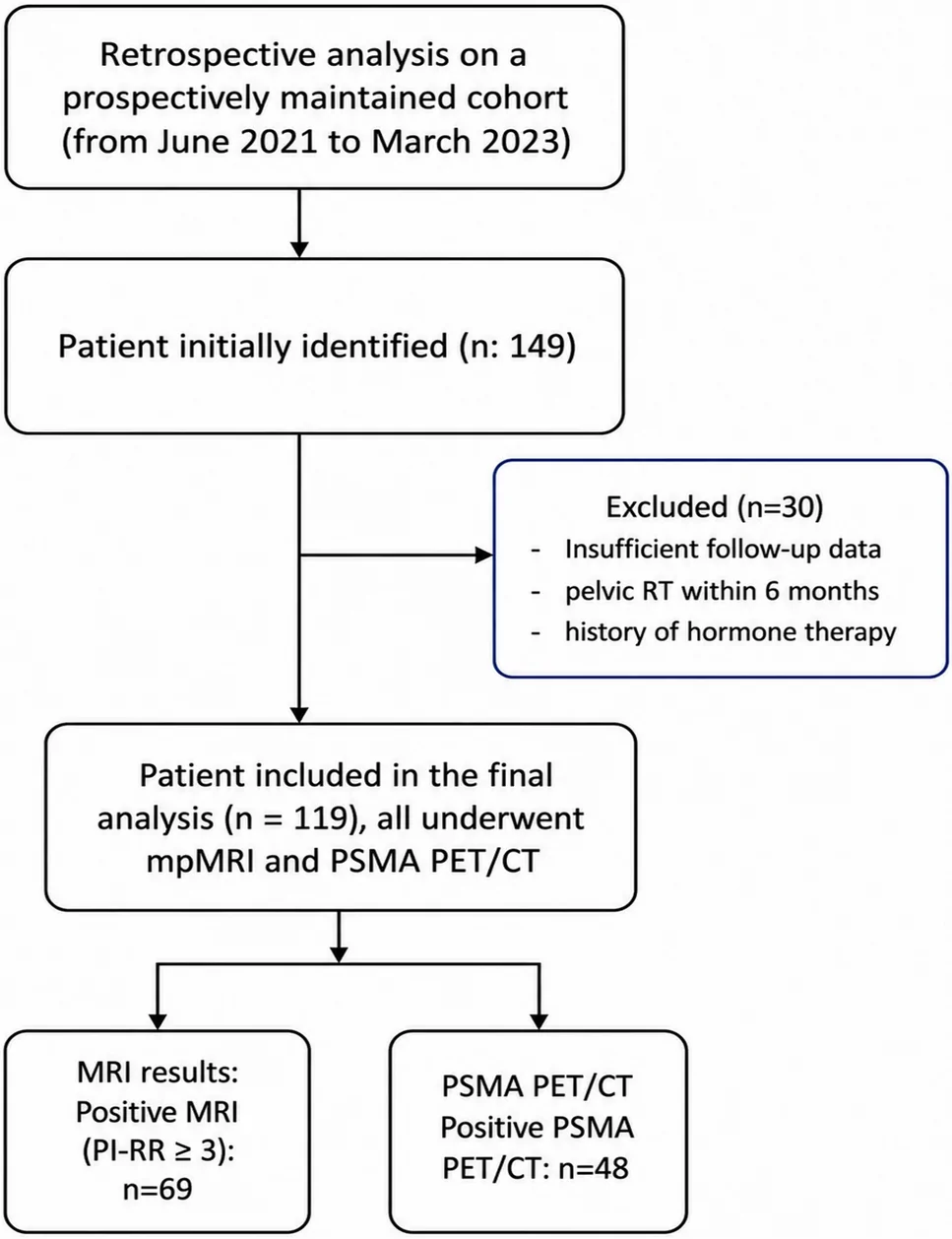
Study flowchart showing the outline of its different phases: enrollment, exclusion criteria, and the final cohort

Overall, 149 consecutive patients with recurrence suspicion after surgery based on clinical factors underwent MRI and PSMA-PET/CT. Of these patients, 30 (20%) were excluded (Fig. 1), with a final sample cohort for the analysis of 119 patients. Median age was 70 years (Interquartile range—IQR—63–75). Median time since initial treatment was 48 months (IQR, 24–84). Median PSA level during relapse was 0.33 ng/ml (IQR, 0.21–0.75). Patients’ characteristics are summarized in Table 1. The median interval between MRI and PSMA-PET/CT was 18 days (interquartile range [IQR]: 10–27 days).

**Table 1:** Summary of cohort population’s clinical, radiological, and pathological data

Characteristic	Population
N. of patients	119
Age, years, *median (IQR)*	70 (63–75)
Pre-biopsy PSA, ng/ml, *median (IQR)*	8.87 (6.36–17.15)
Time since initial treatment (*months*)	48 (24–84)
Pre-treatment ISUP Category, *n (%)*	
1	19 (16%)
2	57 (48%)
3	26 (22%)
4	7 (6%)
5	10 (8%)
Surgical Margin Status, *n (%)*	
R0	90 (76%)
R1	29 (24%)
PSA during relapse, ng/ml, *median (IQR)*	0.33 (0.21–0.75)
EAU risk group for BCR, *n (%)*	
Low risk	71 (60%)
High risk	48 (40%)
Recurrence evidence, *n (%)*	70 (58%)
PI-RR categories, *n (%)*	
PI-RR 1	35 (29%)
PI-RR 2	15 (13%)
PI-RR 3	7 (6%)
PI-RR 4	34 (28%)
PI-RR 5	28 (24%)
PSMA-PET/CT categories, *n (%)*	
Negative	71 (60%)
Positive	48 (40%)
LN on MRI, *n (%)*	
Neg	114 (96%)
Pos	5 (4%)
LN on PET/CT, *n (%)*	
Neg	111 (93%)
Pos	8 (7%)

MRI was positive (PI-RR ≥ 3) in 69 cases (58%); instead, PSMA-PET/CT was found to be positive in 48 cases (40%) (*p* = 0.006). Lymph nodes were scored as positive on MRI and PSMA-PET/CT in 5(4%) and 8(7%), respectively (*p* = 0.392). Among the 119 included patients, histopathologic confirmation was available in 3% (*n* = 4), treatment response (either biochemical or imaging based) in 31% (*n* = 37), follow-up with imaging in 33% (*n* = 39), and clinical-laboratory follow-up in 33% (*n* = 39).

The median duration of imaging follow-up was 12 months (interquartile range, 9–18 months), with a minimum of 24 months required to confirm true-negative findings.

### MRI-based PI-RR assessment and PSMA-PET-CT assessment

The performance of PI-RR in detecting local recurrence, with a score of PI-RR 3 or PI-RR 4 as cutoff for local recurrence detection, is presented in Table 2.

**Table 2:** Diagnostic performance for PCa recurrence detection between MRI (considering a PI-RR score cutoff > 3 and > 4) and PSMA-PET/CT

RP	AUC (95% CI)		SENS (%)	SPEC (%)	PPV (%)	NPV (%)	ACC (%)
MRI	0.87 (0.80–0.93)	PI-RR cutoff					
		> 3	86	78	74	89	81
		> 4	81	86	80	87	84
PSMA-PET/CT	0.62 (0.52–0.72)						
		+	71	52	51	73	63

**Fig. 2:**
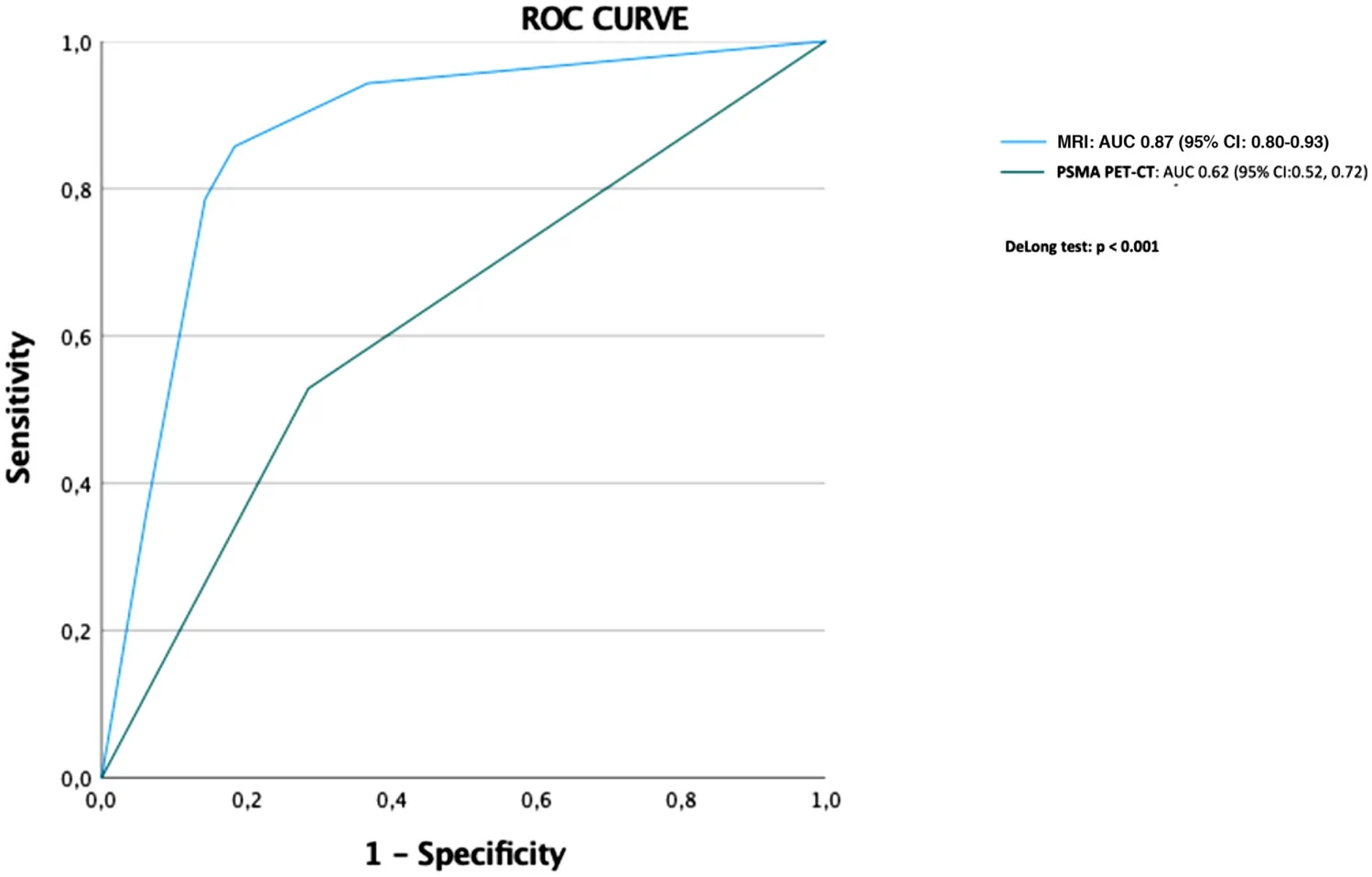
Receiver operating characteristic (ROC) curves comparing the diagnostic performance of MRI and PSMA-PET/CT for the detection of local recurrence after radical prostatectomy. MRI demonstrated a higher discriminative ability (AUC = 0.87; 95% CI: 0.80–0.93) compared with PSMA-PET/CT (AUC = 0.62; 95% CI: 0.52–0.72), with a statistically significant difference between the two curves (*p* < 0.001, DeLong test). The diagonal line represents the line of no discrimination

With a PI-RR score of 3 as cutoff, the sensitivity was 86%, specificity was 78%, PPV was 74%, NPV was 89%, and accuracy was 81%. With a PI-RR score of 4 as cutoff, the sensitivity was 81%, specificity was 86%, PPV was 80%, NPV was 87%, and accuracy was 84%. The AUC was 0.87 (95% CI 0.80, 0.93). Figure 2 shows the receiver operating characteristic curve. The inter-reader agreement was substantial (*k* = 0.64). The performance of PSMA-PET/CT in detecting local recurrence is presented in Table 2.

PSMA-PET/CT sensitivity for recurrence detection was 71%, specificity was 52%, PPV was 51%, NPV was 73%, and accuracy was 63%. The AUC was 0.62 (95% CI 0.52–0.72). Figure 2 shows the receiver operating characteristic curve. The inter-reader agreement was substantial (*k* = 0.81).

### Paired diagnostic performance of MRI and PSMA-PET/CT

MRI correctly identified 59 true-positive and 39 true-negative cases, with 10 false negatives and 11 false positives, corresponding to a sensitivity of 86%, specificity of 78%, and overall accuracy of 81% (95% CI: 74–87%).

PSMA-PET/CT correctly identified 29 true-positive and 30 true-negative cases, with 41 false negatives and 19 false positives (predicted positive *n* = 48), yielding a sensitivity of 71%, specificity of 52%, PPV of 51%, NPV of 73%, and overall accuracy of 63% (95% CI: 54–71%).

The difference in sensitivity between MRI and PSMA-PET/CT was statistically significant (*p* = 0.03, McNemar test), while the difference in specificity approached significance (*p* = 0.06).

Receiver operating characteristic (ROC) analysis confirmed a significantly higher AUC for MRI (0.87, 95% CI: 0.80–0.93) compared with PSMA-PET/CT (0.62, 95% CI: 0.52–0.72; ΔAUC = 0.25, *p* < 0.001, DeLong test).

To further explore discordant findings between the two modalities, a paired reclassification analysis was performed, evaluating the proportion of cases reclassified by one imaging method relative to the other. This analysis aimed to quantify complementary diagnostic contributions of MRI and PSMA-PET/CT, particularly in cases with low PSA levels or equivocal imaging results.

Paired reclassification analysis showed concordant positive findings (MRI + /PET +) in 44 patients (37%) wand concordant negative findings (MRI–/PET–) in 44 (37%), while 25 (21%) were MRI-positive but PET-negative, and 6 (5%) were PET-positive but MRI-negative. Overall, discordant findings were therefore observed in 26% (31/119) of cases, predominantly due to MRI + /PET–results.

### PSA-stratified analysis

A PSA-stratified analysis was performed to further explore the diagnostic performance of MRI and PSMA-PET/CT across different PSA subgroups (< 0.2, 0.2–0.49, 0.5–0.99, and ≥ 1.0 ng/mL).

MRI generally demonstrated higher sensitivity and overall accuracy across all PSA strata, particularly in patients with PSA levels below 0.5 ng/mL. Specifically, at PSA < 0.2 ng/mL, MRI achieved a sensitivity of 82% and an accuracy of 81%, compared with 40% and 61% for PSMA-PET/CT, respectively. Similarly, in the 0.2–0.49 ng/mL subgroup, MRI outperformed PSMA-PET/CT (sensitivity 88% vs. 63%; accuracy 84% vs. 63%).

At higher PSA levels (≥ 0.5 ng/mL), the diagnostic performance of PSMA-PET/CT progressively improved, approaching that of MRI (sensitivity 88% vs. 92%, respectively, in the ≥ 1.0 ng/mL subgroup).A detailed summary of MRI and PSMA-PET/CT performance across PSA subgroups is provided in Supplementary Table 4.

### Clinical and imaging features associated with local recurrence detection

At univariable analysis, the variables significantly associated with prostate cancer recurrence were age (*p* < 0.001), EAU risk grouping (*p* = 0.01), PI-RR assessment with cutoff values of 3 and 4 (*p* < 0.001), and PSMA-PET/CT imaging (*p* < 0.001). Variables with *p* < 0.05 at univariable analysis were subsequently entered into the multivariable logistic regression model. In this model, only age (*p* = 0.002; 95% CI 0.34–0.77) and PI-RR scoring with a cutoff value of 3 (*p* = 0.001; 95% CI 0.19–0.76) remained independently associated with local relapse. The results of both univariable and multivariable analyses are summarized in Table 3.

**Table 3:** Univariable and multivariable regression analysis assessing the correlation with the likelihood of PCa recurrence among clinical and radiological factors

Analysis of patients	Evidence of Recurrence		P*	OR (95% CI)
*n (%)*	Not (49)	Yes (70)		
Age			< 0.001	0.002 (0.34–0.77)
≥ 70	35 (71)	26 (37%)		
< 70	14 (29)	44 (63%)		
PSA at time of recurrence			0.010	0.611 (0.10–0.17)
< 0.33	32 (65)	29 (41)		
≥ 0.33	17 (35)	41 (59)		
ISUP category			0.679	-
= 1	7 (14)	12 (17)		
≥ 2	42 (86)	58 (82)		
Margin status			0.089	-
R0	41 (84)	49 (70)		
R1	8 (16)	21 (30)		
EAU Risk grouping			0.010	0.232 (0.222–0.54)
Low risk	36 (73)	35 (50)		
High risk	13 (26)	35 (50)		
PI-RR, *n (%)*			< 0.001	0.001 (0.195–0.769)
1–2	42 (86)	15 (21)		
3-4-5	7 (14)	55 (79)		
PI-RR, *n (%)*			< 0.001	0.435 (0.172–0.396)
1-2-3	40 (82)	10 (14)		
4-5	9 (18)	60 (68)		
PSMA/PET-CT,			< 0.001	0.483 (0.095–0.199)
Negative	35 (71)	33 (47)		
Positive	14 (29)	37 (53)		

PSA value at initial diagnosis and at relapse, ISUP category, EAU risk grouping, surgical margin status, PI-RR ≥ 4, and PSMA-PET/CT imaging did not show independent predictive value for local recurrence.

## Discussion

The diagnostic performance of MRI and PSMA-PET/CT in detecting prostate cancer recurrence remains a topic of ongoing debate.

In the present study, MRI demonstrated superior accuracy in identifying local recurrence after RP, particularly in patients with low PSA levels (< 0.5 ng/mL). Compared to PSMA-PET/CT, MRI achieved higher sensitivity and negative predictive value (86% vs 71% and 89% vs 73%, respectively), confirming its key role in the early detection of small local relapses that may remain undetectable by molecular imaging. This advantage can be explained by both biological and technical factors. DCE sequence, which represent the dominant sequence in the PI-RR scoring system after RP, allow visualization of early vascular and perfusion-related changes that accompany local tumor regrowth [17, 18], even when PSMA expression is still limited. In contrast, PSMA-PET/CT performance decreases at low PSA values, where tumor volume and receptor density are often insufficient for reliable tracer uptake.

Our analysis highlights that MRI maintains its strong diagnostic performance, particularly in the low-PSA setting, supporting its complementary role to PSMA-PET/CT. MRI is accurate in detecting local recurrence within the prostatectomy bed or seminal vesicle remnants. Therefore, the integration of these techniques should be regarded as synergistic rather than competitive, especially when clinical management decisions rely on accurate localization of recurrence, and it should be based on patients’ risk.

**Fig. 3:**
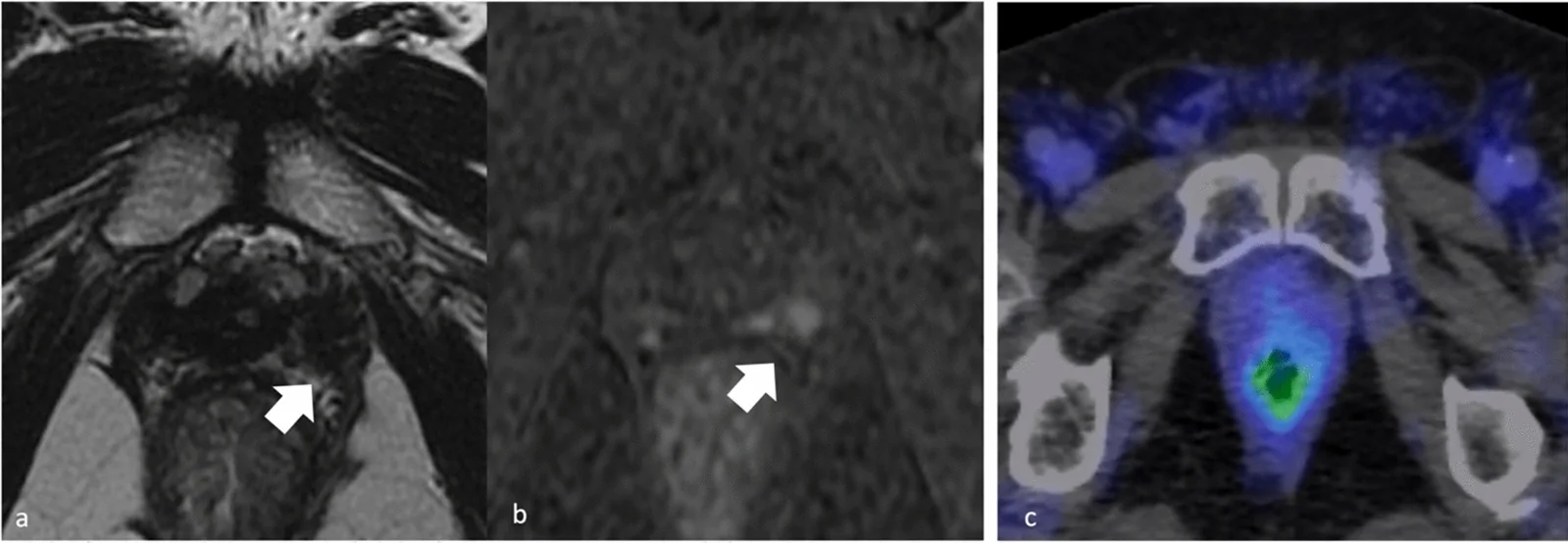
A 64-year-old male patient with BCR after RP (PSA 0.17 ng/mL). **a** T2WI (axial) shows a nodular lesion on the left side of the anastomosis (white arrow); **b** DCE shows a nodular enhancement on the left side of the anastomosis (white arrow); **c** PET/CT image in which there is no evidence of recurrence. MRI is indicative of a PI-RR score 5, since the suspicion focus was on the same site of the primary tumor

Indeed, PSMA-PET/CT can be the first-line imaging modality to be proposed to patients with PSA level of > 0.2 ng/mL, especially when results are likely to influence subsequent treatment decisions; although guidelines recommendations remain weak [2]. Conversely, MRI interpreted using PI-RR scoring system has proven to be an optimal tool for the assessment of the prostatectomy bed or seminal vesicle remnants in detecting local relapse, which are the most common site of tumor recurrence after RP and RT [25, 26, 30] (Fig. 3). Instead, PSMA-PET/CT is the most accurate imaging modality for the detection of metastatic lymph nodes and distant metastasis [11, 31] (Table 4).

**Table 4:** Recommendations for the management of persistent PSA and biochemical recurrence, adapted from European Association of Urology guidelines [2]

EAU Recommendations	Strength rating
PSA recurrence after RP	
Perform a PSMA-PET/CT 1. If PSA level is > 0.2 ng/mL 2. If the result will influence consecutive decision (EAU BCR risk groups)	Weak
Perform a choline PET/CT or fluciclovine PET/CT 1. In case PSMA-PET/CT is not available with a PSA level ≥ 1 ng/mL	Weak
PSA recurrence after RT	
Perform a MR: 1. To localize suspicious areas 2. To guide further biopsies in fit patients for local salvage therapy	Weak
Perform a PSMA-PET/CT (if available) or a choline PET/CT or fluciclovine PET/CT: 1. In fit patients for curative salvage treatment	Strong

Indeed, in addressing the primary aim of this study, our results demonstrate MRI’s stronger performance compared to PSMA-PET/CT, especially in terms of sensitivity and NPV (86% vs 71% and 89% vs 73%, respectively).

Certainly, for patients experiencing BCR after local treatment with curative intent at low risk, in case of positive conventional imaging there is no need of proceeding with next-generation imaging (NGI). Conversely, for patients eligible at low risk for salvage treatments, if conventional imaging is negative, they should be directed to NGI if available, preferably a PSMA-PET/CT scan [32]. On the other hand, patients at high risk should be directed to NGI.

Our results differ significantly from Mingels et al., who reported a PSMA-PET/CT sensitivity and specificity of 0.94 (95% CI:0.81–0.98) and 0.92 (95% CI:0.64–0.99) for local recurrence detection, with a AUC of 0.76 ± 0.05 [33]. This discrepancy may reflect differences in patient cohorts, as our population had a median PSA of 0.33 ng/mL at relapse. Indeed, the literature shows that for PSA value ranging from 0.2 and 0.49 ng/ml 68 Ga-PMSA PET positivity is around 45% (95% CI 39–52) [34], in line with our results. Therefore, the lower accuracy observed for PSMA-PET/CT in this cohort should be interpreted in the context of the low median PSA at relapse (0.33 ng/mL), rather than reflecting the intrinsic diagnostic potential of the technique.

Similarly, on Radzina et al.’ experience, 128 patients with prostate cancer and 32 with biochemical relapse were referred to 68 Ga-PSMA-11 PET/CT, MRI and BS, showing MRI higher diagnostic accuracy in detecting PCa local recurrence, while PSMA-PET/CT resulted to be superior in detecting distant and lymph node metastases [35]. However, it is not possible to properly compare this last result with those presented in the present manuscript, because the number of the events, as LN involvement suspicion, was too small to draw any significant conclusion; indeed, LN resulted positive in 5 cases on MRI and 8 cases on PSMA-PET/CT.

Our data also reinforce previous meta-analysis findings investigating MRI accuracy in detecting PCa local recurrence. This meta-analysis showed how after RP, MRI had a pooled sensitivity and specificity of 82% (95% CI 78–86%) and 87% (95% CI 81–92%), respectively. In the subgroup analysis, compared with T2WI, DCE MRI showed higher pooled sensitivity (85%, 95% CI 78–90%) and specificity (95%, 95%CI 88–99%) [36, 37]. Indeed, DCE MRI was found particularly accurate, and these results support PI-RR recommendations, according to which DCE is the dominant sequence for PCa recurrence detection [17, 38].

Notably, our multivariable regression model confirmed that PI-RR score is the only factor, in addition to age, independently correlating with PCa local recurrence, highlighting the strong role of PI-RR score to standardize MRI acquisition, interpretation and reporting in detecting PCa local recurrence.

Moreover, the robustness of PI-RR is further supported by previous studies in the literature [25, 26], not only the diagnostic accuracy, but also a high inter-observer reliability, even among readers with varying levels of experience.

Recent research has also explored the role of PSMA-PET/MRI as a potential solution to the ongoing debate between MRI and PSMA-PET/CT. In particular, Martinez et al. [39] compared the overall diagnostic performance, presented as detection rate of 68 Ga-PSMA-HBED-CC PSMA-PET/MRI versus MRI, investigating a population of patients with BCR PCa. They found that PSMA-PET/MRI had a significantly higher DR than MRI (*p* = 0.009). However, when assessing the prostatectomy bed, PSMA-PET/MRI revealed only one more focus compared to MRI (*n* = 8;7.01% vs *n* = 7;6.14%). Moreover, MRI indicated several lesions described as equivocal primarily within prostate/prostatic bed [39], suggesting that a standardized approach for MR images interpretation could further improve MRI accuracy. A meta-analysis further confirmed the high diagnostic performance of PET/MRI with a pooled detection rate of 80.9% at restaging [40]. However, availability and cost might limit the applicability of this approach in clinical practice. Moreover, differences between our findings and previous studies reporting higher PSMA-PET/CT performance may be partly explained by variations in PSA distribution, tracer characteristics, and interpretation criteria across cohorts. In the recent meta-analysis by Zhang et al. [41], PSMA-PET/CT demonstrated a higher overall positivity rate than mpMRI for detecting biochemical recurrence (0.68 vs. 0.56), but with marked heterogeneity attributed to differences in PSA levels, radiotracer type (^68 Ga-PSMA-11 vs. ^18F-based compound), and reader experience.

Taken together, our findings advocate for a pragmatic, risk- and PSA-stratified approach to imaging in biochemical recurrence. Based on our findings and current guideline recommendations [2], MRI, particularly when interpreted using the PI-RR system, should represent the first-line imaging modality in low- to intermediate-risk patients, given its high sensitivity and NPV for local recurrence. Conversely, PSMA-PET/CT should be prioritized in high-risk patients, or when PSA levels are higher, owing to its superior capability in detecting nodal and distant disease.

Ultimately, MRI and PSMA-PET/CT should be regarded as complementary tools within an integrated diagnostic strategy. Their sequential or combined use, guided by PSA kinetics, clinical risk, and institutional resources, can maximize diagnostic accuracy and optimize personalized management of patients with recurrent prostate cancer.

The following limitations need to be acknowledged.

First, this was a retrospective analysis of prospectively enrolled patients, which may introduce selection bias and limit causal inferences. Second, the reference standard was based on a composite truth (including imaging follow-up and clinical outcome), which may have introduced partial verification bias, since not all patients underwent histopathological confirmation.

Third, the relatively high prevalence of recurrence in our cohort (58%) may have influenced predictive values, particularly PPV and NPV, which are inherently dependent on disease prevalence.

Fourth, all image evaluations were performed by experienced urogenital radiologists, with a substantial agreement, which may not reflect routine clinical practice and thus can limit the generalizability of our findings to less specialized/less-experienced settings. MRI and PSMA-PET/CT examinations were performed in two different centres; however, each modality was consistently acquired at a single site using standardized protocols and identical scanner settings, minimizing the potential impact of inter-site variability on diagnostic performance.

In addition, it should be noted that standardized PET/CT scoring systems were not applied in this study.

Moreover, another limitation of our study is that in patients with multiple lesions, only the lesion with the highest PI-RR score was considered for analysis. Although this approach was intended to focus on the lesion most likely to represent site of recurrence, it may have introduced a selection bias.

Finally, cost-effectiveness and access analyses were not included, which limits the assessment of the broader clinical and economic impact of the proposed diagnostic approach.

## Conclusion

MRI assessment of the prostatectomy bed using the PI-RR scoring system demonstrates higher accuracy for detecting local prostate cancer recurrence, particularly in patients with low PSA levels after radical prostatectomy. Conversely, PSMA-PET/CT remains the preferred modality for identifying nodal and distant disease. A tailored, PSA-stratified diagnostic pathway should be adopted: MRI may be used primarily to rule out local recurrence in patients with favorable prognostic profiles, whereas PSMA-PET/CT should be employed to rule in local or systemic recurrence in those with less favorable features. Prospective studies are warranted to validate these findings and refine patient selection for each imaging modality.

## Data Availability

The authors confirm that the data supporting the findings of this study are available within the article and its supplementary materials.

## Abbreviation:

MRI: Magnetic resonance imaging
PSMA-PET/CT: Prostate-specific membrane antigen positron emission tomography/computed tomography
PCa: Prostate cancer
RP: Radical prostatectomy
RT: Radiation therapy
BCR: Biochemical recurrence
PSA: Prostate-specific antigen
CT: Computed tomography
NCCN: National comprehensive cancer network
EAU: European association of urology
PI-RR: Prostate imaging for recurrence reporting
EANM: European association of nuclear medicine
SUVmax: Maximum standardized uptake value
GS: Gleason score
ISUP: International society of urological pathology
PPV: Positive predictive value
NPV: Negative predictive value
AUC: Area under the curve
T2WI: T2-weighted imaging
DWI: Diffusion-weighted imaging
DCE: Dynamic contrast enhancement
CI: Conventional imaging
NGI: Next-generation imaging
